# Editorial: Global excellence in cardiovascular and smooth muscle pharmacology: north and Central America

**DOI:** 10.3389/fphar.2023.1326266

**Published:** 2023-11-20

**Authors:** Prasanth Puthanveetil, Layla Al-Nakkash, Arinola O. Lampejo, Walter L. Murfee

**Affiliations:** ^1^ Department of Pharmacology, College of Graduate Studies, Midwestern University, Downers Grove, IL, United States; ^2^ Department of Physiology, College of Graduate Studies, Midwestern University, Glendale, IL, United States; ^3^ J. Crayton Pruitt Family Department of Biomedical Engineering, University of Florida, Gainesville, IL, United States

**Keywords:** cardiovascular, smooth muscle, blood, pharmacology, therapeutic targets, cell signaling

Cardiac and vascular tissue are two intrinsically intertwined parts of the circulatory system that directly influence each other in response to changes in functional and metabolic behavior. To further explore the crosstalk between these two systems, we present this Research Topic on the role certain agents, such as drugs, metabolic regulators, and genetic diseases, play in altering cardiovascular health. This series also focuses on how alterations in the activity of specific drug targets like enzymes, receptors, and mRNA affect cardiac and vascular health. Blood serves as the connecting link between the cardiac and vascular system, with components of both blood and blood pressure participating in cardiac-to-vascular crosstalk. This series has specifically focused on complications and associated targets in cardiac tissue, the vascular system, and the blood. In this Research Topic, we have considered this triad together to highlight novel areas of cardiovascular research and to provide insight into the complex connections between this trio of systems.

In this research series, Menon et al. demonstrated the influence of iron on determining drug fate through the mediation of drug transporters. This study has high relevance with regards to anti-cancer treatment, cardiac toxicity, and mineral (iron) imbalance. The authors demonstrate that iron overload diminishes expression and activity of ABCB8 (an efflux transporter), which is primarily involved in ejecting the anti-cancer drug doxorubicin (DOX) from cardiac cells. This work highlights the adverse effect of anti-cancer drugs on cardiac tissue and reveals the important role the efflux transporter ABCB8 has as a major mediator in bringing about this homeostasis. This work also portrays how a systemically abundant mineral like iron can influence drug-induced cardiac toxicity. The authors use H9C2 cardiomyocytes to demonstrate the influence of iron loading and iron deficiency on ABCB8 expression and the resulting effect on DOX retention and toxicity in the cardiomyocytes. To validate their hypothesis, authors also utilized a mouse model of hereditary hemochromatosis (HH), a genetic model of iron overload. This unique approach expounds upon the significance of iron overload, which significantly increases doxorubicin toxicity. Overall, this work clearly underlines a novel connection between iron, the ABCB8 efflux transporter, and DOX-induced cardiac cell toxicity.

In the study by Menon et al. a novel Vascular Health Index (VHI) is introduced to elucidate how different disease conditions and drugs might impact vascular health conditions associated with metabolic disease. Motivated by the recognition that underlying diseases like hypertension, hyperglycemia, hyperlipidemia, and oxidative stress conditions can drastically impact vascular health, this study also explores the effects of therapeutic interventions like anti-hypertensives, anti-diabetics, hypolipidemic drugs, and antioxidants on long term complications. VHI includes structural and functional readouts including microvessel density, stress vs. strain relationships, and acetylcholine-based vascular reactivity in both gracilis muscle arterioles and middle cerebral arteries. Mass, glucose, insulin, and TNF-α level measurements are also incorporated. The findings demonstrate that plasma, insulin, and TNF-α levels have a strong, negative correlation with the VHI that was evident across all intervention groups. Additionally, lower insulin resistance and lower systemic inflammation was accompanied by a higher (or better) VHI. This work emphasizes the potential significance of adopting an early aggressive treatment strategy for prevention of end organ damage. The work also emphasizes the idea that the use of single pharmacological agent to target risk factors will probably have minimal beneficial outcomes, thus promoting the utilization of either multiple agents or the pleiotropic effects of a single agent.

The work by Ihunnah et al. introduces another example of cell-cell interactions across systems. In their study, the authors revealed how targeting red blood cell alterations associated with sickle cell disease can influence microvascular function. They found that targeting the nuclear factor erythroid 2-like 2 (Nrf2) activator via CDDO-Methyl Ester administration is a therapeutic strategy for sickle cell disease and is accompanied by a decrease in nitric oxide (NO) mediated vasodilation. Human microvascular endothelial cells (HMVECs) and human erythroleukemia cells were used to study the cytoprotective function of the drug candidates dimethyl fumarate (DMF) and CDDO-Methyl (CDDO-Me) in an *in vitro* modeling system, while pulmonary arteries from Townes Sickle Cell Anemia mice (both males and females) were used to model the effects of CDDO-Methyl ester administration *in vivo*. Using these models, the authors were able to reveal the mechanism behind Nrf2-mediatied activation preventing NO-mediated vasodilation is mediated through endothelin receptors (ETA and ETB). CDDO-Methyl Ester mediated Nrf2 activation was accompanied by an enhanced ETA and ETB expression.

Finally, the review article included in the Research Topic by Ren et al. focuses on myocardial fibrosis which is an underlying commonality in many cardiovascular complications. The authors detail the use of both modern western medicine and traditional Chinese medicine in the treatment of myocardial fibrosis. While they express that traditional Chinese medicine is effective at treating myocardial fibrosis, they also explain that there is a need for more rigorous scientific testing and elucidation of underlying mechanisms that potentially make traditional Chinese medicine so effective. Thus, clarity regarding the impact of these specific active components on drug metabolizing enzymes, is needed to promote the widespread development and plausibility of traditional Chinese medicine. Altogether, this Research Topic of four articles provides readers with in-depth examples that represent the breadth of research required to consider how cardiovascular relationships impact basic pathophysiology and therapeutic design.

This collection of four articles in this Research Topic have highlighted various areas in cardiovascular and smooth muscle pharmacology, along with therapeutics for blood diseases that impact the cardiovascular system. To conclude, the published articles in this series will provide readers in depth information in diverse areas of cardiovascular science.

